# Altered metabolomic states elicited by Flg22 and FlgII-28 in *Solanum lycopersicum*: intracellular perturbations and metabolite defenses

**DOI:** 10.1186/s12870-021-03200-5

**Published:** 2021-09-21

**Authors:** Dylan R. Zeiss, Paul A. Steenkamp, Lizelle A. Piater, Ian A. Dubery

**Affiliations:** Research Centre for Plant Metabolomics, Department of Biochemistry, University of Johannesburg, P.O. Box 524, Auckland Park, 2006 Johannesburg, South Africa

**Keywords:** Tomato, Flagellin, Plant defence, Metabolic profile, Plant-microbe interactions

## Abstract

**Background:**

Surveillance of potential pathogens is a key feature of plant innate immunity. For non-self-recognition plants rely on the perception of pathogen-derived molecules. Early post-perception events activate signaling cascades, leading to the synthesis of defense-related proteins and specialized metabolites, thereby providing a broad-spectrum antimicrobial coverage. This study was concerned with tracking changes in the tomato plant metabolome following perception of the flagellum-derived elicitors (Flg22 and FlgII-28).

**Results:**

Following an untargeted metabolomics workflow, the metabolic profiles of a *Solanum lycopersicum* cultivar were monitored over a time range of 16–32 h post-treatment. Liquid chromatography was used to resolve the complex mixture of metabolites and mass spectrometry for the detection of differences associated with the elicitor treatments. Stringent data processing and multivariate statistical tools were applied to the complex dataset to extract relevant metabolite features associated with the elicitor treatments. Following perception of Flg22 and FlgII-28, both elicitors triggered an oxidative burst, albeit with different kinetic responses. Signatory biomarkers were annotated from diverse metabolite classes which included amino acid derivatives, lipid species, steroidal glycoalkaloids, hydroxybenzoic acids, hydroxycinnamic acids and derivatives, as well as flavonoids.

**Conclusions:**

An untargeted metabolomics approach adequately captured the subtle and nuanced perturbations associated with elicitor-linked plant defense responses. The shared and unique features characterizing the metabolite profiles suggest a divergence of signal transduction events following perception of Flg22 vs. FlgII-28, leading to a differential reorganization of downstream metabolic pathways.

**Supplementary Information:**

The online version contains supplementary material available at 10.1186/s12870-021-03200-5.

## Background

In general, plants are resistant to most pathogens, being protected by the innate immune system comprising of several defensive layers. However, most phytopathogens have developed specialized mechanisms of compromising the immune system, leading to infection and host death. The first layer consists of pre-formed barriers such as the cuticle, cell wall and constitutively produced antimicrobial compounds that function to maintain cellular integrity, provide structural support and prevent pathogen entry [1]. Pathogens that surpass the physical barriers contend with the second layer i.e., host recognition system. This sophisticated surveillance system is used to distinguish ‘self’ from ‘non-self’ interactions and detect opportunistic phytopathogens within the surrounding environment [2, 3]. This molecular crosstalk between the host plant and the pathogen determines the eventual outcome of the plant-pathogen interaction.

The recognition of M/PAMPs (microbe- or pathogen-associated molecular patterns) is a key event during plant-pathogen interactions that is required for the induction of the plant defense response through pattern-recognition receptors (PRRs) [4, 5]. The first plant MAMP-receptor pair, namely the FLAGELLIN-SENSING 2 (FLS2) receptor-like kinase (RLK) along with its ligand, the 22-amino acid flagellin epitope, Flg22, has become the best-described example of pathogen-triggered immunity (PTI) in plants [5, 6]. Perception and direct binding of the Flg22 epitope initiates a cascade of signaling events, i.e., the interaction of FLS2 with the co-receptor protein BAK1 (brassinosteroid insensitive 1-associated receptor kinase 1, the Arabidopsis orthologue of SERK3A/3B, *Solanum lycopersicum* somatic embryogenesis receptor kinase 3 A/ 3B). These interactions result in the rapid influx of calcium ions, the synthesis of ROS and the activation of mitogen-activated protein kinase (MAPK) cascades [4, 7]. This triggers a complex defense response which includes FLS2*-*directed stomatal closure that interrupts pathogen progression, plant cell wall reinforcement and the expression of pathogenesis-related *(PR)* defense genes that culminate to restrict pathogen growth [8]. Evolutionary divergence has resulted in various plant species recognizing different peptide sequences present in bacterial flagellin [8].

Most land-based plants can perceive the epitope of Flg22, however, recent advances have demonstrated that plants from a subset of the Solanaceae family, e.g. tomato, potato and pepper can recognize an additional epitope of flagellin, termed FlgII-28, and that this perception occurs independently of FLS2 [4, 5]. FlgII-28 recognition is attributed to a newly described receptor called FLAGELLIN-SENSING 3 (FLS3) that mediates plant immunity, thereby enhancing resistance to the bacterial pathogen [5]. The FLS3 receptor represents an alternative mechanism of flagellin perception and, therefore, the expression of this solanaceous-specific PRR in crop plants normally unable to detect FlgII-28 could be utilized to confer resistance to pathogens that have evolved methods of evading Flg22 detection [5]. The degree to which the ligand – receptors interactions promote immune responses, as well as the similarity in molecular mechanisms involved, remains largely unknown.

During the initial stages of interaction, the M/PAMP molecules are perceived by the host’s specialized membrane bound immune receptors resulting in the activation of several intracellular signaling cascades as well as downstream defense strategies capable of subverting pathogen invasion [2, 9, 10]. The activation of these signaling cascades (Fig. S1) results in physiological modifications to the host, which may define a resistant, tolerant, or susceptible phenotype. Metabolic processes, as final recipients of biological information flows and thus indicators of the biological phenotype, are integral components of the induced plant defense response and function in both physiological and biochemical disease resistance. Untargeted profiling provides an opportunity to evaluate the applications of metabolomics in studying elicitor-mediated metabolic fluctuations that occur in *S. lycopersicum*. Similarly, it allows a method of characterizing the magnitude of metabolic reprogramming that occurs after perception and early signaling events and, subsequently, whether the signals from different elicitor treatments produce convergent or divergent downstream effects [11]. Research conducted on plant diseases and conserved molecular patterns assists in elucidating the fundamental aspects of microbial pathogenesis and the extent to which plants respond to the threat [12]. Consequently, such research has significance and allows the downstream development of effective and sustainable methods of disease control.

## Results

During the experimental procedures, the tomato cultivar ‘Star 9001’ was treated with the Flg22 and FlgII-28 peptides – MAMP components of the flagella, also regarded as pathogen-derived elicitors. This cultivar was chosen due its apparent disease resistance towards a number of pathogens [13] as well as minimal venation patterns present on the abaxial side of the leaves, which eased the process of pressure infiltration. Consequently, the study was performed to determine: whether an untargeted metabolomics platform can distinguish between Flg22- vs. FlgII-28-induced perturbations to the metabolome; and which metabolite classes and metabolic pathways function in defense signaling and induced responses following elicitor perception.

### Reactive oxygen species production and oxidative burst

**Fig. 1:**
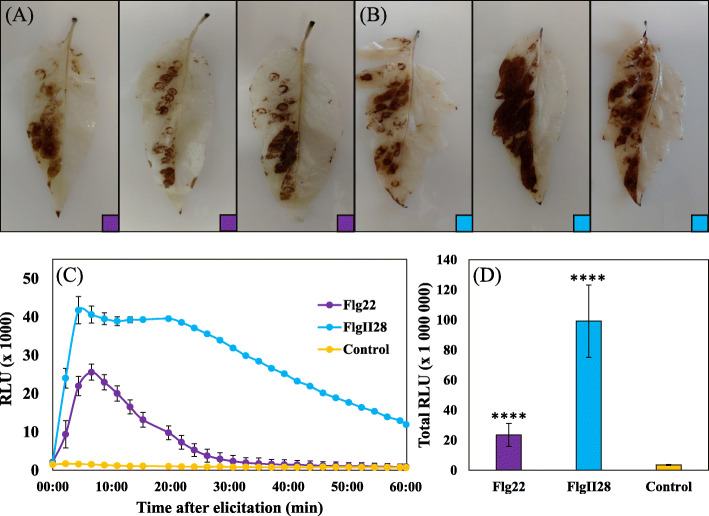
Induced oxidative burst in tomato leaves after treatment with the Flg22 (purple) and FlgII-28 (blue) peptide elicitors. **Top** The elicitor responses visualized using the histochemical stain, 3,3-diaminobenzidine. The left side of the leaves were treated with **(A)** Flg22 or **(B)** FlgII-28 while the right side served as a water control. **Bottom** The elicitor responses visualized using the luminescence assay. **C** The kinetics of reactive oxygen species (ROS) production over 60 min, described as ∑ relative luminescence units (RLU). **D** The total ROS production over 60 min shown as the integrated area under the curve. A pairwise Student’s *t*-test was performed to compare the elicitor treatments with the control where the asterisks indicate levels of statistical significance (**** = *P* ≤ 0.0001)

During the DAB staining protocol, the left abaxial side of the leaves was treated with each respective elicitor, while the right abaxial half was supplied with the water control. The brown color product (Fig. 1A, B) reveals the presence of H_2_O_2_ and associated reactive oxygen species (ROS), signatory of initial plant immune responses. The time-dependent production of ROS in response to Flg22 and FlgII-28 elicitation is corroborated by the luminescence assay curves (Fig. 1C, D), indicating differences in the timing and extent of the ROS generation by the two elicitors. The luminescence data confirms that of the DAB staining, highlighting the ability of the Star 9001 cultivar to perceive the flagellin-derived elicitors.

### Chromatographic and mass spectrometric analyses of tomato leaf extracts

The analyses of the Flg22 / FlgII-28 elicitor-treated tomato leaves were conducted as an untargeted metabolomics study to obtain all the statistically significant biomarkers that contribute to the changes in the metabolome throughout the period of the study. The UHPLC-MS base peak intensity (BPI) chromatograms of data generated in negative ESI mode of the leaf extracts are presented as Figs. S2 and S3. Shown are the representative MS chromatograms obtained from extracts prepared after 16 h, 24 and 32 h following infiltration. The chromatograms highlight metabolic variations as a result of time-dependent elicitor treatment over a retention time (Rt) window of 25 min. Qualitative differences are reflected by the peak intensities where the *y*-axis represents the relative peak intensity of the *x*-axis metabolites at their respective Rts (min). Variation in peak intensities and presence or absence of peaks reflect differential changes to the metabolomes.

### Data analysis and statistical modeling

**Fig. 2:**
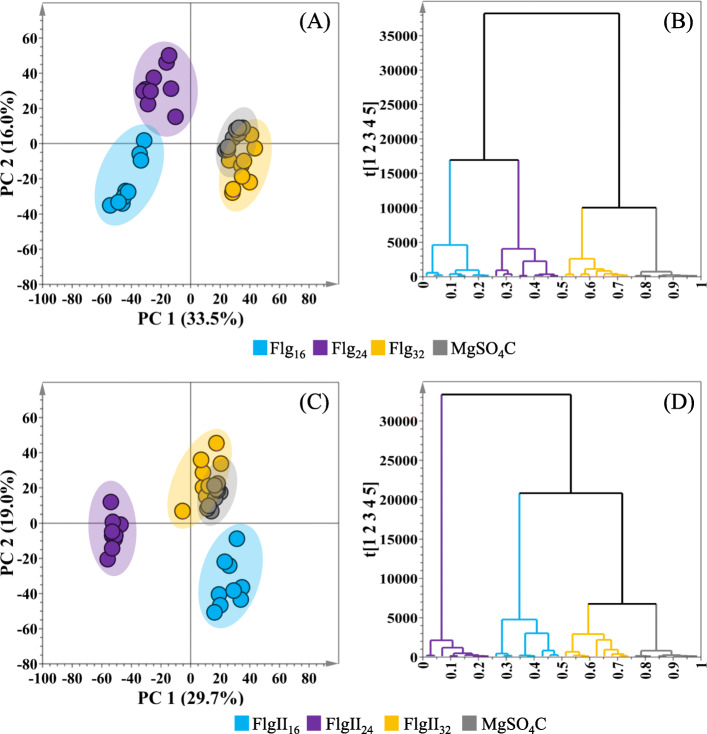
Principal component analysis (PCA) and hierarchical cluster analysis (HiCA) models of UHPLC-MS ESI(–) data of extracts from tomato leaves. The PCA score plots illustrate the group clustering after treatment with **(A)** Flg22 peptide and **(C)** the FlgII-28 peptide, and incubated for 16 h (blue), 24 h (purple) and 32 h (yellow) respectively. A MgSO_4_ control (grey) was included in each elicitor treatment. The hierarchical cluster dendrograms are derived from the PCA model data and outline the overall hierarchical structure of **(B)** Flg22 and **(D)** FlgII-28 treatment over the above-mentioned incubation times

Multivariate data analysis (MDVA) was performed to sort through the data matrices and reveal trends in the metabolome over the time intervals, as well as to detect similarities/ differences in the metabolite profiles of the plants in response to the flagellin peptide treatments. From a complex dataset, PCA (a descriptive method that provides a global and qualitative visual representation of similarity or dissimilarity between and within samples) was used to explore the data and discover possible group clusters, trends, or sample outliers. As an unsupervised projection-based statistical tool, PCA allows for an exploratory analysis by projecting the original multidimensional data matrix in a lower-dimensional space (Fig. 2A, C), thus permitting the extraction and summarization of underlying group trends in a visual manner, finally displaying the systematic variation present in the data [14–18]. PCA trends were further examined by hierarchical cluster analysis (HiCA), a complementary method of data exploration, as it can reveal trends within the data that may be hidden within the principal components of PCA. HiCA is based on Ward’s linkage method, considering distance clusters between- and within-samples [14]. The computed HiCA models (Fig. 2B, D) served to evaluate subgroupings present within the data set and depicted major distinct groupings corresponding to various time points. As such, both PCA and HiCA assisted in evaluating the overall structure of the data sets, revealing underlying patterns, trends and subgrouping (time- and treatment-dependent groupings). These observations highlight aspects of the biochemical phenomena (altered metabolomic states) attributed to tomato responses to the two flagellin elicitors.

In addition, the data matrices were subjected to supervised statistical modeling, i.e. OPLS-DA, as a (multivariate) binary classification and for variable selection method (as described in the Methods section). OPLS-DA is an extension of PLS-DA, a regression method, featuring an integrated orthogonal signal correction (OSC)-filtering method; where the variance of interest (e.g. the flagellin-treatments) is separated from the variance that is not related (orthogonal) to the defined Y-block variables (i.e. treated vs. control / hypothesis). This provides interpretational and discriminatory benefits, as the variance important for the defined group classification is focused into a single predictive component, and variance that is not related to the tested hypothesis is computed into orthogonal components. This makes it easier to link the information of interest (i.e. that which focused into a single component) to the experimental variables (e.g. metabolite features), as well as assessing the predictive power of a sub-set of discriminatory makers [15, 16, 18].

**Fig. 3:**
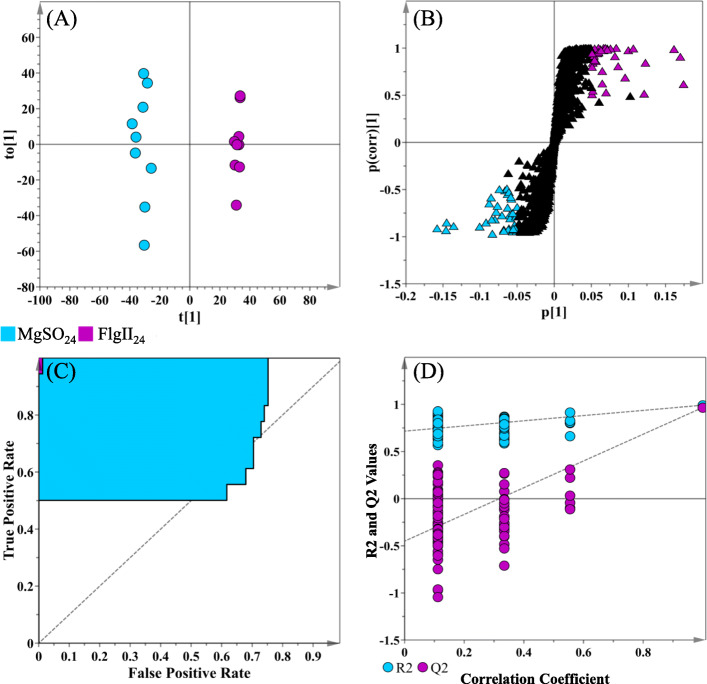
An orthogonal projection to latent structures discriminant analysis (OPLS-DA) model for data processing of leaf extracts of the MgSO_4_ control vs. FlgII-28 treatment at the 24 h incubation. **A** Scores plot showing group separation of control vs. treated (FlgII_− 24_ - purple vs. MgSO_4 − 24_ - blue). The calculated model yielded R^2^X (cum) = 54.5 %, R^2^Y (cum) = 99.2 % and Q^2^ (cum) = 96.7 %. The goodness-of-fit parameters for the OPLS model, R^2^X and R^2^Y, represent the fraction of the variance of the x and y variable explained by the model, while Q^2^Y suggests the predictive performance of the model. Model validation by 7-fold cross-validated analysis of variance (CV-ANOVA, [19]) was statistically significant with *p*-value *=* 1.636 × 10^− 9^. **B** The corresponding OPLS-DA loadings S-plot. Variables at the extremes of the loadings S-plot (|p(corr)| of ≥ 0.5; |(p1)| ≥ 0.05) represent possible discriminating variables. **C** A receiver operating characteristic (ROC) curve summarizing the selective ability of the binary classifier (S-plot), with a classifier having a perfect discrimination (top left corner) to indicate 100 % sensitivity and specificity. **D** The response permutation test plot (*n* = 100) for the OPLS-DA model

OPLS-DA models were constructed to inform on class separation, and subsequently, to select discriminant ions (variables) positively correlated to each of the elicitor treatments at the selected time intervals. A set of representative figures summarizing the OPLS-DA model, receiver operating characteristic (ROC) curve and permutation plot corresponding to the data sets of the FlgII-28 elicitor treatment at 24 h post-elicitation and corresponding MgSO_4_ control, is presented in Fig. 3A-D. A seven-fold cross-validation (CV) method was applied as a tuning procedure in computing the chemometric models. The parameters and metrics necessary for evaluating model quality, such as R^2^X(cum), R^2^Y, Q2(cum) and CV-ANOVA calculated *p-*values, were recorded for each of the computed supervised models and are presented in Table S1.

The OPLS-DA score plot (Fig. 3A) showed class separation with distinct sample clustering between the MgSO_4_ control and FlgII-28 experimental condition. The corresponding OPLS-DA loadings S-plot (Fig. 3B) selected discriminating features deemed statistically significant with positive correlations towards the elicitor treatment at the defined time interval. The S-plot permits a visual interpretation of the OPLS-DA model by revealing metabolite features (*m/z* ions) that contribute to the class separation in Fig. 3A. Discriminant features with a |p(corr)| of ≥ 0.5 and a co-variance value of |(p1)| ≥ 0.05 were selected for further analysis using MS spectral-based metabolite identification. The significance for the observed group separation was measured by calculating the CV-ANOVA *p*-values, applying *p* < 0.05 as a cut-off [19]. The *p*-values of each computed supervised model are tabulated for both Flg peptides (Table S1). A ROC curve (Fig. 3C) assessed the performance of the OPLS-DA model in terms of selectivity, showing that the computed model, as a binary classifier, had perfect discrimination with regards to sensitivity and specificity [20]. The predictive capabilities of the OPLS-DA model were validated with the use of a response permutation test (with *n* = 100), shown in Fig. 3D [20, 21]. The permutation test showed that the models have higher calculated R^2^ and Q^2^ values in comparison to the 100 model permutations, concluding that the obtained OPLS-DA model was statistically superior to the generated permutated models. The R^2^ and Q^2^ values from all the permutated models for all conditions were tabulated for comparative purposes (Table S1). The equivalent set of figures for Flg22 is presented as Fig. S4.

### Metabolite profiling and relative quantification of annotated metabolites

From the OPLS-DA S-plot, individual metabolite features, within the selection parameters described in the experimental procedure, were subjected to descriptive statistics, i.e., the calculation of control and treatment averaged peak intensities, the standard deviation, *p*-values, and coefficient of variation. An aggregate of 34 metabolites were putatively identified from the tomato leaf tissue (Table 1 and Table S2). Many of the metabolites described in Table 1 have been previously reported either within the tomato plant itself or in related species within the Solanaceae family [13, 17, 22–25]. The annotated metabolites are listed according to Rts with corresponding *m/z* values. From the analyzed data matrices, nine metabolite classes were identified which included: amino acid derivatives, lipid species, steroidal glycoalkaloids, hydroxybenzoic acids, hydroxycinnamic acid (HCA) derivatives, flavonoids and hydroxycinnamic acid amides (HCAAs).

**Table 1:** Annotation of discriminatory metabolites from tomato leaf tissue displaying a positive correlation towards the flagellin-derived elicitor treatments (Flg22 and FlgII-28) after 16 h, 24 and 32 h time intervals. Differential accumulation of metabolites are shown in Table S2

#	Rt (min)	*m/z*	Putative identification	Chemical formula	Error (ppm)
1	1.42	371.059	Caffeoyl glucaric acid	C_15_H_15_O_11_	-8.0
2	3.22	285.058	Genistate xylopyranoside	C_12_H_13_O_8_	-12.5
3	3.45	397.167	Benzoyl ornithine glycoside	C_18_H_25_N_2_O_8_	13.5
4	4.13	658.154	Glutathionyl-caffeoyl quinic acid	C_26_H_32_N_3_O_15_S	-1.7
5	4.70	431.153	Benzyl alcohol dihexoside	C_19_H_27_O_11_	-6.6
6	4.95	353.085	Caffeoyl quinic acid	C_16_H_17_O_9_	-7.9
7	5.30	353.084	Caffeoyl quinic acid	C_16_H_17_O_9_	-10.7
8	5.51	367.158	Dihydroxy dimethoxy prenylchalcone	C_22_H_24_O_5_	7.9
9	6.40	401.140	Benzoyl alcohol pentose glycoside	C_18_H_25_O_10_	-13.2
10	6.79	385.110	Sinapoyl glycoside	C_17_H_21_O_10_	-10.4
11	7.52	387.163	Hydroxyjasmonic acid glycoside	C_18_H_28_O_9_	-7.8
12	8.38	476.155	Unknown	C_26_H_24_N_2_O_7_	-8.1
13	8.63	367.100	Feruloyl quinic acid	C_17_H_20_O_9_	-9.3
14	9.37	296.061	Benzoyl oxindole acetic acid	C_17_H_14_NO_4_	-10.9
15	9.86	245.090	Acetyl tryptophan	C_13_H_13_N_2_O_3_	-12.9
16	10.35	344.112	Feruloyl noradrenaline	C_18_H_18_NO_6_	-5.6
17	10.74	298.107	Coumaroyl dopamine	C_17_H_17_NO_4_	-4.9
18	10.99	444.165	Coumaroyl tyramine glycoside	C_17_H_17_NO_3_	20.6
19	11.20	460.160	Unknown	C_23_H_26_NO_9_	-2.8
20	11.21	609.145	Rutin	C_27_H_30_O_16_	-1.8
21	11.75	490.170	Feruloyl dopamine glycoside	C_20_H_29_NO_13_	27.3
22	12.97	328.117	Feruloyl dopamine	C_15_H_20_NO_8_	1.9
23	13.00	349.183	Acetyl feruloyl agmatine	C_17_H_25_N_4_O_4_	-14.6
24	13.73	282.112	Coumaroyl tyramine	C_17_H_16_NO_3_	-5.5
25/6	13.81	677.282	Unknown	C_27_H_49_O_19_	-7.8
27	14.20	312.121	Feruloyl tyramine	C_18_H_18_NO_4_	-10.0
28	14.40	453.231	Phosphatidyl glycerol (14:1(9Z)/0:0)	C_20_H_38_O_9_P	11.2
29	14.59	1076.520	Dehydrotomatine + FA*	C_51_H_82_NO_23_	-7.7
30	14.83	1078.560	α-Tomatine + FA*	C_51_H_83_NO_23_	14.8
31	15.02	423.184	Phosphatidic acid (8:0/8:0)	C_19_H_37_O_8_P	15.7
32	15.20	447.220	Unknown	C_21_H_35_O_10_	-7.9
33	15.25	423.221	Phosphatidic acid (8:0/8:0)	C_19_H_37_O_8_P	13.4
34	15.32	495.255	Palmitoyl-glycero-phosphatidyl serine	C_22_H_43_NO_9_P	-14.3
35	16.25	495.257	Palmitoyl-glycero-phosphatidyl serine	C_22_H_43_NO_9_P	-16.6
36	16.30	581.281	Phosphatidyl inositol (17:2(9Z.12Z)/0:0)	C_26_H_46_O_12_P	13.3
37	16.72	327.214	Trihydroxyoctadecadienoic acid	C_18_H_31_O_5_	-11.2
38	16.82	333.188	Hydroxydecanoic acid rhamnoside	C_16_H_29_O_7_	-11.6
39	17.41	329.231	Trihydroxyoctadecenoic acid	C_18_H_33_O_5_	-7.1

Metabolites were annotated in ESI(–) mode using UHPLC–MS. The metabolite features were annotated according to level 2 of the Metabolomics Standards Initiative (MSI)

*Abbreviations: FA = formic acid adduct

**Fig. 4:**
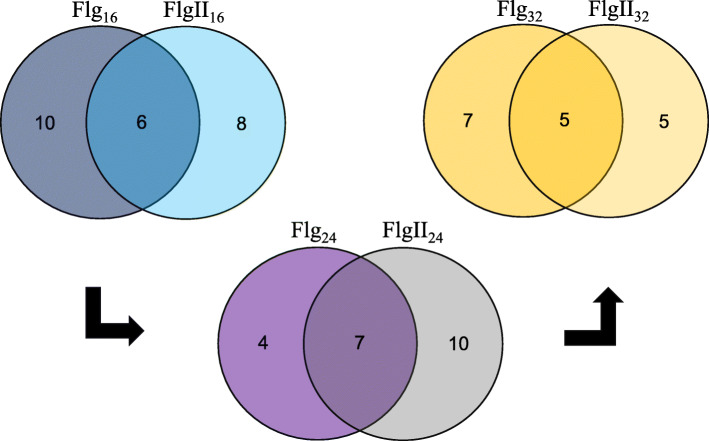
Venn diagrams displaying the partial overlap of *m/z* ion features present in tomato leaves, deemed statistically significant and positively correlated to the flagellin-derived elicitor treatments. The features were selected from the OPLS-DA models generated from the 16 h (blue), 24 h (purple) and 32 h (orange) individual datasets comparing the Flg22 (Flg) and FlgII-28 (FlgII) treatments with the MgSO_4_ control. The numerical values depict the metabolite features (listed in Table 1) that are unique to the Flg22 vs. FlgII-28 treatments over the time intervals, and conversely, that overlap in the treatments

Venn diagrams were constructed, based on the discriminant ions listed in Table 1, to compare the presence/ absence of metabolites at time intervals of 16, 24 and 32 h post-elicitation (Fig. 4). These highlight the overlapping patterns of features unique as well as shared between the two elicitor treatments over the time intervals. The Flg22 and FlgII-28 treatments display a high level of overlapping similarity in the metabolite content positively correlated to the treatments at the specified time intervals. The generated data from Table 1; Fig. 4 revealed an average of six metabolites positively correlated to both elicitor treatments which would suggest that similar metabolic pathways were activated and utilized following perception of the two peptides. The general trend observed from the Venn diagrams would suggest that the end-result of Flg22 vs. FlgII-28 elicitation differs with regards to the timing of the responses which may be related to the differences in the respective profiles of the initial oxidative bursts.

**Fig. 5:**
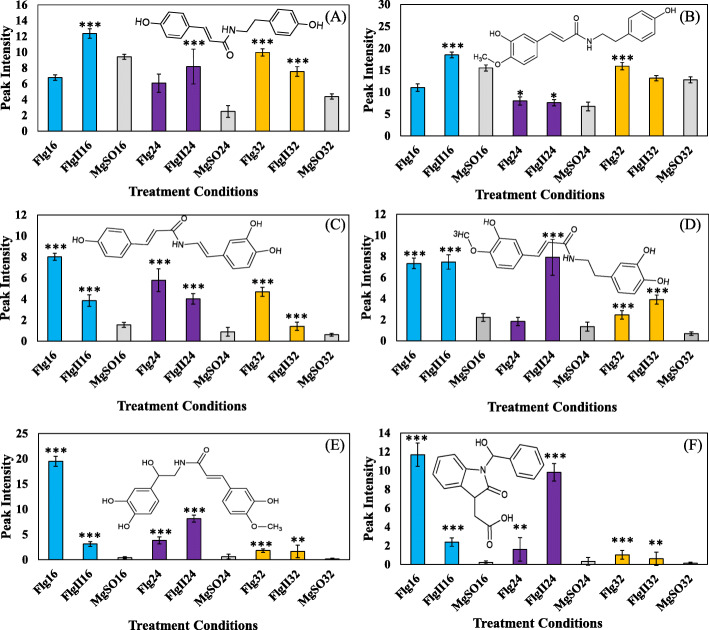
Fluctuating cellular levels of phenolic amide metabolites involved in metabolome changes in tomato leaves elicited by Flg22 vs. FlgII-28. The figures show **(A)** coumaroyl tyramine, **(B)** feruloyl tyramine, **(C)** coumaroyl dopamine, **(D)** feruloyl dopamine, **(E)** feruloyl noradrenaline and **(F)** benzoyl oxindole acetic acid in response to Flg22 (Flg) and FlgII-28 (FlgII) elicitor treatments at the 16 h (blue), 24 h (purple) and 32 h (orange) time intervals. A MgSO_4_ control (MgSO - grey) was included for each time point. Each data bar is presented as a mean value ($\stackrel{-}{x}$ of *n* = 3 × 3 *=* 9 samples) with the error bars indicating the calculated standard deviation (σ). A two-condition paired Student’s *t*-test was performed to compare the treatments with the MgSO_4_ control where the asterisks indicate levels of statistical significance (* = *P* ≤ 0.01, ** = *P* ≤ 0.001, *** = *P* ≤ 0.0001)

The relative peak intensities of metabolite signatures overlapping in both elicitor treatments (Fig. 5, at 16, 24 and 32 h) were further analyzed. Several compounds from the hydroxycinnamic acid amide (HCAA) class were found to overlap in the elicitor treatments. The cellular levels of coumaroyl tyramine were found to increase over the time trial intervals in the FlgII-28 elicitor treatment (Fig. 5A). A delayed response for the afore-mentioned metabolite was observed in the Flg22 treatment. Regarding the FlgII-28 treatment, levels of feruloyl tyramine were found to increase during the 16 h interval followed by a cellular decrease to homeostasis at the 24 and 32 h intervals (Fig. 5B). Conversely, the levels of feruloyl tyramine only started to increase at the 24 h interval increasing to the 32 h interval, during the Flg22 treatment (Fig. 5B). The cellular levels of coumaroyl dopamine increased at the 16 h interval and remained elevated through the latter intervals for both elicitor treatments (Fig. 5C). Similarly, levels of feruloyl dopamine were elevated during the 16 and 32 h time intervals for both treatments (Fig. 5D). At the 24 h stage, the cellular levels of feruloyl dopamine decreased in response to Flg22 treatment while also revealing a cellular increase in the FlgII-28 treatment. The relative levels of both feruloyl noradrenaline (Fig. 5E) and benzoyl oxindole acetic acid (Fig. 5F) increased throughout the time intervals in response to the elicitor treatments.

**Fig. 6:**
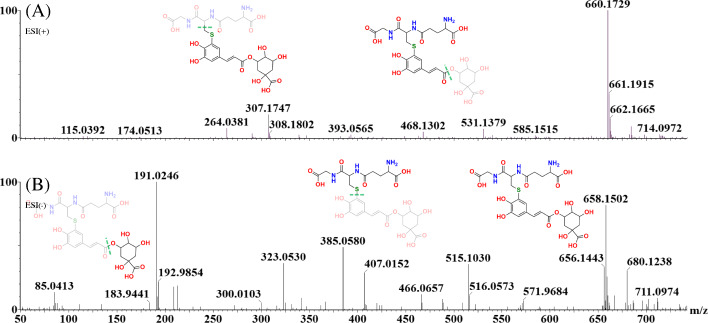
The mass spectral fragmentation pattern of glutathionyl-*S*-caffeoylquinic acid in **(A)** positive and **(B)** negative ionization modes. The MassFragment™ software facilitated structural elucidation and compound identification, using the spectral patterns in both ionization modes. In ESI(–), the precursor ion is 658.15 [M-H]^−^, while the main fragment ions are 515.10 [M-H-C_5_H_10_N_2_O_3_]^−^, 385.05 [M-H-C_10_H_16_N_3_O_6_]^−^ and 191.02 [M-H-C_19_H_22_N_3_O_9_S]^−^. In ESI(+) mode, the precursor ion is 660.17 [M + H]^+^, while the main fragment ions are 468.13 [M + H-quinic acid^+^] and 307.17 [M + H-C_16_H_17_O_9_]^+^. These fragments correspond to what has been described in [26]

**Fig. 7:**
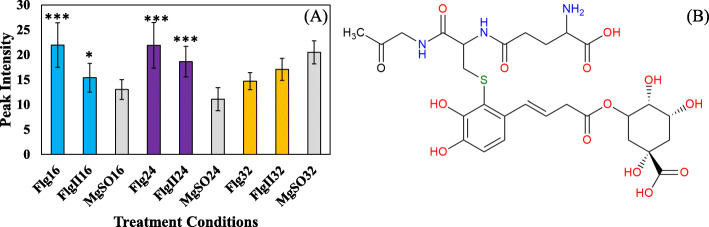
A bar graph indicating the fluctuating cellular levels of the glutathionyl-*S*-caffeoylquinic acid conjugate, a discriminatory marker in the changing metabolome of tomato leaves responding to flagellin-derived peptides. **A** The relative abundance of glutathionyl-*S*-caffeoylquinic acid after Flg22 (Flg) and FlgII-28 (FlgII) treatments at the 16 h (blue), 24 h (purple) and 32 h time (orange) time intervals. A MgSO_4_ control (MgSO - grey) was included for each time point. Each data bar is presented as a mean value ($\stackrel{-}{x}$ of *n* = 3 × 3 *=* 9 samples) with the error bars indicating the calculated standard deviation (σ). A two-condition paired Student’s *t*-test was performed to compare the treatments with the MgSO_4_ control where the asterisks indicate levels of statistical significance (* = *P* ≤ 0.01, ** = *P* ≤ 0.001, *** = *P* ≤ 0.0001). **B** The chemical structure of the putatively annotated glutathionyl-*S*-caffeoylquinic acid conjugate

A novel metabolite conjugate of *S. lycopersicum*, glutathionyl-*S*-caffeoylquinic acid, was detected as a feature positively correlated to both the peptide treatments. The mass spectral analysis of glutathionyl-*S*-caffeoylquinic acid is provided in both ionization modes to illustrate how the MassFragment plugin of the MassLynx software facilitated the annotation of the fragment ions and verified the overall mass fingerprint (Fig. 6; Table 2). The elemental composition of each fragment was calculated as a secondary method of validating compound structural identity. In both Flg22 and FlgII-28 treatments the glutathione conjugate revealed a cellular increase at the 16 and 24 h time intervals (Fig. 7A). At the 32 h interval the cellular levels of the conjugate decreased, returning to a new homeostatic level.

**Table 2:** The LC-MS^2^ spectral and UV-Vis analysis indicating compound characteristics of the glutathionyl-*S*-caffeoylquinic acid conjugate extracted from *S. lycopersicum*

Chemical formula	HR-ESI-MS found	HR-ESI-MS calculated	Mass difference	ppm error	MS^2^ of 660.17 [M + H]^+^	UV-Vis max (nm)
C_26_H_34_N_3_O_15_S^+^	660.1693 [M + H]^+^	660.1705 [M + H]^+^	-0.0012	-1.8	660.17 [M + H]^+^ 585.15 [M-H-C_2_H_4_NO_2_]^+^ 530.15 [M + H-C_5_H_8_NO_3_]^+^ 468.13 [M + H-quinic acid]^+^ 307.17 [M + H-C_16_H_17_O_9_]^+^	283, 519
					**MS**^**2**^** for 658.15 [M-H]**^**-**^	
C_26_H_32_N_3_O_15_S^−^	658.1548 [M-H]^−^	658.1559 [M-H]^−^	-0.0011	-1.7	658.15 [M-H]^−^ 515.10 [M-H-C_5_H_10_N_2_O_3_]^−^ 466.07 [M-H-quinic acid]^−^ 385.05 [M-H-C_10_H_16_N_3_O_6_]^−^ 191.02 [M-H-C_19_H_22_N_3_O_9_S]^−^	

The fragments (*m/z* ions) obtained overlap with what has been described in scientific literature [26]

## Discussion

Plant immune receptors recognizing pathogen-derived molecules are part of an intricate sensing and multi-layered signaling network. Experimental treatments with live bacterial pathogens would expose the plant to several different MAMPs, each with a unique PRR and associated perception events able to trigger downstream signal transduction [13]. Previous studies of plant metabolomes have shown that treatment with single elicitors result in subtle metabolic perturbations, in comparison to the cocktail of MAMPs associated with live pathogen treatment. Tomato has become a favored species for metabolomics research, filling a niche not occupied by Arabidopsis [27]. Tomato varieties exhibit a natural variation in their responsiveness to Flg22 and FlgII-28 [28]. Flg22 is recognized by FLS2 and FlgII-28 by FLS3, with the latter representing a possible orthogonal means for flagellin perception in the Solanaceae [5]. Where ROS are generated upon PAMP perception, changes occur in the redox balance within cells, leading to oxidative stress and signal transduction events. Variation in signal transduction networks determines perceived inputs and consequent defense outputs and may thus potentially result in an altered response at the metabolome level. To investigate differential responses to Flg22 and FlgII-28 tomato leaves (Star 9001 cultivar) was treated with the flagellum-derived peptides and responses tracked over a time interval ranging from 16 to 32 h post-elicitation.

### Flagellin-induced oxidative burst, production of reactive oxygen species

Plants from the Solanaceae have evolved more specialized and sensitive mechanisms of perceiving a second epitope in bacterial flagellin [8, 28]. The semi-quantitation of ROS indicated that the FlgII-28 elicitor triggered a more intense and sustained response in the tomato leaf disks compared to that of Flg22. This observation broadly corresponds with that reported for *S. pimpinellifolium* [5] and *S. lycopersicum*, cv. Rio Grande [29]. It appears that the FlgII-28 extended ROS response is conserved in all solanaceous species that respond to FlgII-28 [29].

Elicitor perception induces ROS production by NADPH oxidases belonging to the respiratory burst oxidase homolog (RBOH) family [30]. The outcome is dependent on the concentrations of ROS generated and the extent of the immune response. At high concentrations ROS may function as triggers of programmed cell suicide or have direct anti-microbial activity, while at moderate concentrations may serve as signaling molecules to activate downstream immune outputs in signaling loops [31]. The tight regulation of ROS is essential to maintaining the redox balance within the system. Several antioxidant mechanisms are activated to return cellular redox levels to homeostasis to avoid detrimental effects on host cells. Accordingly, the timing, the extent and the location of ROS production is important for its role in stress signaling and plant redox-dependent immune responses.

Following elicitor perception, signal transduction events generated from the dynamic interactions between the FLS2 and FLS3 receptors and their respective ligands may converge or diverge, potentially creating a different functional outcome at the metabolome level. Previous studies of the components involved in FLS2 and FLS3 signaling in tomato have reported some parallels with regards to mitogen-activated protein kinase (MAPK) activation and gene expression as immunity outputs [5, 32]. Relatedly, a more comprehensive report that included ROS production and whole-plant responses to infection by *Pseudomonas syringae* pv. *tomato* concluded that there may be some differences in the molecular signaling between these two flagellin-sensing receptors in tomato [29].

To gain deeper insights into the metabolic footprint of upstream recognition events of Flg22 and FlgII-28, an untargeted metabolomics approach was followed for the generation of biochemical data correlated to the tomato plant defense responses against the two bacteria-derived MAMPs. A combined total of 34 metabolites positively correlated to the elicitor treatments were annotated. Here it was observed that the flagellin-derived elicitors lead to the production of similar secondary metabolites that, in turn, perform similar metabolic roles. However, Venn diagrams indicated a differential response with regards to shared and unique secondary metabolites as identified from OPLS discriminant analyses. Table 1 lists nine metabolite classes that include: amino acids, lipid species, steroidal glycoalkaloids, hydroxybenzoic acids, flavonoids, HCA derivatives and HCA amide conjugates. While correlation does not imply causality [33], an overview of the functional roles that these metabolites might play in the altered metabolomic state was previously presented [13]. A secondary metabolite class that was a prominent feature of the altered metabolomes resulting from the flagellin elicitor treatments were the HCAAs, and these are discussed below. Additionally, a novel elicitor-induced glutathionyl conjugate of caffeoylquinic acid (a chlorogenic acid) was detected in *S. lycopersicum*, suggesting a link between redox signaling events and accumulation of phenolic antioxidants.

### Signatory metabolites – the hydroxycinnamic acid amides

**Fig. 8:**
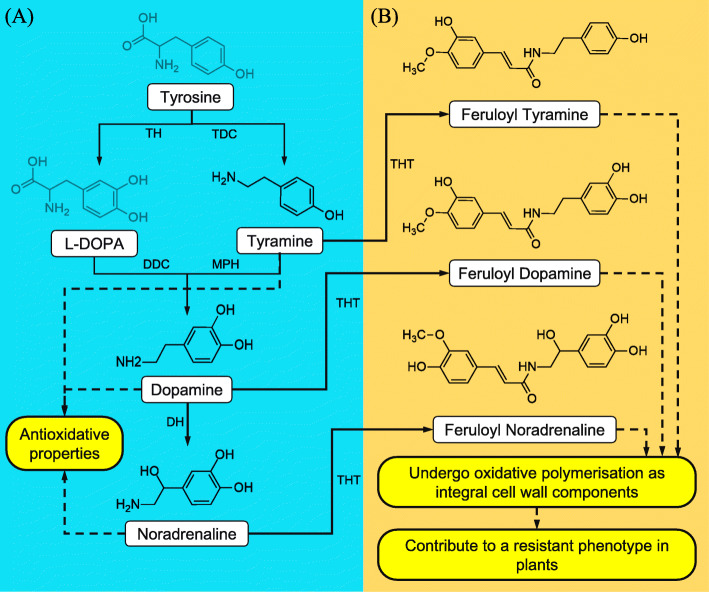
Schematic presentation of tyrosine metabolism leading to the production of hydroxycinnamic acid amides (HCAAs). **A** Tyramine and the two catecholamines have been shown to have high antioxidant properties [34]. **B** Each compound shown in the pathway may be conjugated to hydroxycinnamic acid (HCA) derivatives (e.g., ferulic acid as shown). The most common conjugates include coumaric- and ferulic acid. Several sources have described the incorporation of the HCAAs into the cell wall [36, 37]. Compounds that were not detected as discriminant ions due to the flagellin elicitor treatments are lightly shaded i.e., tyrosine and L-DOPA. Dashed arrows indicate multiple enzymatic reactions leading to the production of a specific compound. Abbreviations: TH, tyrosine hydroxylase; TDC, tyrosine decarboxylase; DDC, dopa decarboxylase; MPH, monophenol hydroxylase; DH, dopamine hydroxylase; THT, tyramine *N-*(hydroxycinnamoyl) transferase

The flagellin elicitor treatments resulted in the production of several tyrosine-derived compounds, with the most notable being tyramine, dopamine, and noradrenaline (Table 1; Fig. 8). These molecules have similar chemical structures and are sequential within a catalytic pathway [34]. The decarboxylation of tyrosine leads to the production of tyramine which, in turn, is hydroxylated to form dopamine (3,4-dihydroxyphenylalanine) – that can further be hydroxylated leading to the formation of noradrenaline (Fig. 8). Dopamine itself has demonstrated strong antioxidant properties with reducing activity comparable to that of glutathione, ascorbic acid, quercetin and luteolin [35]. The increased production of the nitrogen-containing molecules during the flagellin peptide treatments could suggest a method of quenching the defense-induced ROS observed during the perception assays (Fig. 1). It should be noted that many of the above-mentioned metabolites were discovered as conjugates of HCAs (e.g. coumaric- and ferulic acid) (Table 1), that alter the physicochemical properties of the molecules and bestow additional cellular roles. These tyrosine derivatives fall into the aromatic amine (AA) compound class and do form associations with other metabolites – leading to the bioconversion in a conjugated state.

The HCAAs are the combination of the AAs conjugated to various HCAs, and have been previously reported in tomato and other plant species from the Solanaceae family [35, 38, 39]. HCAAs are also produced as phytoalexins of *Oryza sativa* during pathogen infection and are responsive to hormones associated with plant defense [40]. The aromatic compounds can participate in cell wall reinforcement via peroxide-mediated crosslinking, by acting as direct antioxidant- and antibacterial agents, and depending on chemical structure, may be degraded leading to the production of hydrogen peroxide to complete the cycle (Fig. 8). The detection of the HCAAs in the flagellin elicitor treatments (Flg22 and FlgII-28) points to the ability of the MAMPs to induce metabolic alterations in support of plant defense comparable to pathogen treatment. The variance observed in the metabolite profiles following treatment indicates that, from a metabolomic standpoint, a divergence of signal transduction events exists following perception of Flg22 and FlgII-28 as elicitors, leading to a differential reorganization of downstream metabolic pathways. The level of elicitor-induced metabolic reprogramming that takes place is proportionally linked to the impact of the initial perceived signal. This would suggest that the FlgII-28 elicitor induces a more intense and prolonged metabolic response. The HCAAs can be regarded as the final accumulated products of AA metabolism, often serving as a cellular storage form to regulate the overall metabolic pool of the parental constituents [35].

It should be noted that literature linking specific AAs to biotic stress is scarce and the knowledge conceptualizing the functional roles of these compounds is still not fully elucidated. The discovery of the described compounds at the various time points reveals that flagellin-elicitor perception results in the activation of the AA biosynthetic pathway, as well as the specialized transferase enzymes required for the conjugation to phenolic acids. This would suggest a functional role of the HCAAs not only in downstream plant defense, as observed with pathogen infection, but also in the early processes after elicitor perception.

### Glutathione, redox signaling, and redox homeostasis

Oxidative stress results from the complex chemical and physiological imbalance between free radicals and antioxidants within a biological system [41], e.g. during the de novo synthesis of ROS as a component of stress signaling in plant defense responses [41–43]. Redox homeostasis and antioxidant signaling serves as a metabolic interface between stress perception and physiological responses [44]. Plants maintain the redox homeostasis through dedicated pathways that produce several antioxidant molecules (such as the HCAs and associated conjugates and derivatives) and enzymes to protect against radical-mediated damage to cellular components [41].

The process of cellular toxicity is mitigated by large numbers of ROS quenching proteins e.g. superoxide dismutase, ascorbate peroxidase and peroxiredoxin as well as non-enzymatic molecules including ascorbic acid, glutathione, flavonoids and HCAs with associated derivatives [43, 45]. Glutathione (γ-glutamyl-cysteinyl-glycine - GSH) has multiple functional roles due to its ability to participate in thiol-disulphide interactions, being continuously oxidized to a disulphide form (GSSG) that reverts back to GSH by the action of NAPDH-dependent glutathione reductase enzymes [46]. Functioning in combination, ascorbic acid and glutathione form the foundations of the Asada-Halliwell cycle, a dedicated pathway of ROS detoxification that serves the conservation of homeostatic levels of ascorbic acid and glutathione and results in the effective detoxification of free radical species [44, 45, 47, 48].

Research supports the ability of GSH to conjugate with chlorogenic acids *in vivo* (Figs. 6 and 7), with the biosynthesis of the complex molecule being attributed to a functional role in plant defense responses [26, 49]. Interestingly, this is the first reported case of the glutathionyl-*S*-caffeoylquinic acid conjugate in the metabolome of *S. lycopersicum*. Studies have shown that glutathionylation increases compound polarity and provides an additional ligand modification that is recognized by transporter proteins for cross membrane movement [26]. Glutathionylation has been suggested to facilitate the transport of phenylpropanoids into vacuoles and into the apoplastic space [50]. Glutathione conjugation, in general terms, is regarded as a detoxification reaction, but in some cases may present as a storage form for later bioactivation of the initial parent compound, e.g. as potentially for chlorogenic acids [51]. Glutathione-*S*-transferase (GST) catalyzed reactions have also been suggested to be associated with the synthesis of the above-mentioned conjugate as an intermediate of downstream biosynthetic processes. Both GSH and the chlorogenic acids have an antioxidant capacity bestowing them the ability of alleviating oxidative stress.

Linking the GSH conjugate with a functional role in plant defense, the primary suggestion would be the attenuation of oxidative stress occurring in the biological systems during the elicitor treatments. The production of GSH in the chloroplasts and peroxisomes at early stages of plant pathogen interactions has been linked to increased resistance [52]. Jasmonic acid (JA), but not hydrogen peroxide, has been shown to lead to the expression of several GSTs required for GSH synthesis [53]. Additionally, the GST enzymes have shown to be auxin-inducible, can interact with indole acetic acids (Table 1) as non-substrate ligands and participate in hormone transport [54].

## Conclusions

An untargeted metabolomics approach was used to detect metabolite signatures overlapping in the Flg22 and FlgII-28 elicitor treatments of tomato. The metabolites identified by multivariate statistical modeling were derived from both primary and secondary metabolism. The metabolite features positively correlated to the elicitor-treatments could be categorized into the hydroxybenzoic acids, HCAs and associated derivatives, HCA conjugates like HCAAs, steroidal glycoalkaloids, flavonoids and lipid metabolite classes. Other miscellaneous signatures, such as amino acid derivatives and phytohormones (e.g. a jasmonic acid derivative), were also detected.

The observed trend in the metabolite profiles at different time points revealed plausible evidence that the flagellin elicitor treatments resulted in the activation of tyrosine metabolic pathways – leading to the production of HCAA compounds (including coumaroyl tyramine, feruloyl tyramine, coumaroyl dopamine and feruloyl dopamine), benzoyl conjugated indole-containing compounds, as well as lipid species recently linked with early immune signaling processes. Although the pathogen-induced production of the HCAAs has been previously reported, this is believed to be the first metabolomic approach cataloguing the single elicitor-induced production of these compounds in *S. lycopersicum*. HCAA compounds, in conjunction with their described antioxidant- and antimicrobial activities, may also participate in cell wall reinforcement. However, the functional role of the HCAAs in plant defense, linking molecules derived from phenylalanine deamination and tyrosine decarboxylation, is still to be fully conceptualised.

Additionally, a glutathionyl-S-caffeoylquinic acid conjugate, not previously reported in tomato, was produced in leaf tissue at the 24 h time point of the elicitor treatments, suggestive of a role limiting potential oxidative stress/damage resulting from events occurring during MAMP perception and early immune responses. Finally, the untargeted metabolomics approach was successful in capturing the subtle perturbations associated with elicitor-linked plant defense responses. Tracking the 16–32 h post-treatment trend in the metabolite profiles revealed that the two flagellin-derived MAMPs are differentially perceived as elicitors by tomato plants, with both producing oxidative burst responses, but dissimilar in intensity and kinetics, subsequently leading to nuanced differences in downstream metabolic profiles.

## Methods

### Plant cultivation

Seeds of the commercially available tomato cultivar, ‘Star9001’ were obtained from Stark Ayres, Pty. Ltd. (Bredell, South Africa, www.starkeayres.co.za) and cultivated in germination mixture (Culterra, Muldersdrift, South Africa). The plants were grown under greenhouse conditions: a light/dark cycle of 12 h/12 h, with the light intensity set at 80 µmol/m^2^/s and the temperature regulated to between 22 and 24 ^o^C. We confirm that relevant institutional, national, and international guidelines and legislation were adhered to in the research.

### Flagellin peptides

The peptide elicitors were synthesized at ≥ 90 % purity. The Flg22 peptide (GL Biochem, Shanghai, China) with sequence QRLSTGSRINSAKDDAAGLQIA was previously described [28], as was the FlgII-28 peptide (GenScript, Piscataway, NJ, USA) with sequence ESTNIQRMRELAVQSRNDSNSATDREA [5, 28]. Stock solutions of each peptide elicitor were made to 1 mg/mL and used as diluted samples during the various experiments.

### 3,3’-Diaminobenzidine (DAB) histochemical staining for detection of ROS

Leaves of mature tomato plants were treated with the flagellin-derived elicitors and stained with a 3,3’-diaminobenzidine (DAB, Sigma-Aldrich, St. Louis, MO, USA) solution to visualize the presence of hydrogen peroxide (H_2_O_2_). The protocol was performed with slight modifications as previously described [55]. Briefly, the abaxial side of the leaves were treated with 1 µM Flg22 or 1 µM FlgII-28 by means of pressure infiltration and incubated for 30 min. Care was taken to avoid excess wounding or mechanical damage during pressure infiltration. One h prior to the experiment, the DAB solution (1 mg/mL in water, pH 3.8) was prepared. The bottle was covered in aluminum foil due to light sensitivity and placed on a heating block at 50 ^o^C to solubilize. The leaves were excised from the plant and placed in the DAB solution under light at 23 ^o^C for 8 h with constant agitation. After the incubation period, the leaves were removed from the DAB solution and immersed in boiling 70 % ethanol for 10 min. After cooling, the leaves were transferred into an absolute ethanol solution at room temperature and left overnight. The visible, brown polymerized precipitate in the host tissue was produced as a product of the reaction between DAB and H_2_O_2_.

### Luminescence assay for kinetics of ROS production

Leaf disks (0.4 cm^2^) were punched out from fully expanded leaves using a cork borer. The leaf disks were floated adaxial side up on 200 µL MilliQ water in a 96-well microtiter plate (Nunc, Roskilde, Denmark) which was placed under light at room temperature for 24 h. After the incubation period the water from each well was completely removed and replaced with a 100 µL of a master mix solution composed of 34 µg/mL luminol (Sigma-Aldrich, St. Louis, MO, USA) and 20 µg/mL horseradish peroxidase (Sigma-Aldrich, St. Louis, MOUSA) and each of the respective elicitors (1 µM Flg22, 1 µM FlgII-28) in water. Special consideration was taken to limit mechanical damage of the leaf disks during the disk floating and water removal steps. The luminescence was measured every 2 min for 60 min using a Synergy HT Biotek microplate reader (Biotek Instruments, Winooski, VT, USA). The data was exported to an Excel file for further analysis. To account for natural variability 3 leaf disks per plant were taken, where a total of 24 leaf disks were used per treatment condition.

Univariate statistical analysis: The kinetics of reactive oxygen species production over time, described as ∑ relative luminescence units were analyzed based on the integrated area under the curve. A pairwise Student’s *t*-test was performed to compare the elicitor treatments with the control.

### Plant elicitation

The Flg22 and FlgII-28 elicitors were diluted to a 500 nM concentration. The tomato plants were watered generously 5 h prior to elicitor inoculation to open leaf stomata and facilitate inoculation. The plants were treated with the solutions by pressure infiltration into the leaves using a blunt-ended syringe. An 8 mM MgSO_4_ control was included. It should be noted that the fragility and complex reticulate venation inherent with tomato leaves complicate the inoculation process and that care should be taken to avoid/limit wounding or mechanical damage. Following inoculation, the plants were incubated for 16 h, 24 and 32 h respectively. After each incubation time the inoculated leaves were harvested from three different plants, quenched in liquid nitrogen, and stored at -80 ^o^C until further use. The experimental design included three biological replicates, generated for each elicitor treatment at each time point, and analyzed in triplicate, generating *n* = 9 required for metabolomics investigations [56].

### Metabolite extraction for metabolomics analyses

Frozen leaf tissues were pulverized with a pre-cooled mortar and pestle. Two grams of pulverized material were extracted with 80 % methanol in a 1:10 (w/v) ratio. The samples were sonicated twice in a sonicator bath for 30 min at 20 ^o^C. Cell debris was pelleted with a bench top swinging-bucket centrifuge set at 5525 x*g* and 5 ^o^C for 20 min. The supernatants were evaporated to 1 mL using a rotary evaporator at 55 ^o^C, carefully transferred into 2 mL microcentrifuge tubes and dried in a heating block overnight at 55 ^o^C. The samples were then reconstituted in 500 µL of 50 % liquid chromatography-grade methanol: MilliQ water solvent (1:1, v/v), and filtered through 0.22 μm nylon syringe filters into chromatography vials fitted with 500 µL inserts prior to being stored at 4 ^o^C until analysis.

### Ultra-high performance liquid chromatography coupled to high definition mass spectrometry (UHPLC–HDMS)

Two µL of each sample extract was analysed in triplicate on an UHPLC-high definition quadrupole time-of-flight high-definition MS (UHPLC–qTOF–HD–MS) system equipped with an electrospray ionization (ESI) source (SYNAPT G1, Waters Corporation, Manchester, UK). The analytes were separated on an Acquity HSS T3 reverse-phase column (2.1 × 150 mm x 1.7 μm; Waters Corporation, Milford, MA, USA) using a binary solvent system consisting of acetonitrile (Romil Chemistry, Cambridge, UK): MilliQ water, with both solvents containing 0.1 % formic acid (FA, Sigma-Aldrich, Munich, Germany) and 2.5 % isopropanol (Sigma-Aldrich, Munich, Germany). A gradient elution method was used over a 30 min run with a flow rate set to 0.4 mL/min. The elution was started at 2 % (v/v) acetonitrile from 0 to 1 min, raised to 70 % acetonitrile from 1 to 22 min, taken up to 95 % from 22 to 23 min then kept constant at 95 % acetonitrile from 23 to 26 min. The composition of the mobile phase was then reverted to 2 % acetonitrile from 26 to 27 min, for column cleaning and equilibration from 27 to 30 min. The chromatographically separated metabolites were detected with the aid of a SYNAPT G1 HDMS (Waters Corporation, Manchester, UK) set to acquire data in both positive and negative ionization modes. The MS conditions were as follows: capillary voltage of 2.5 kV, sample cone voltage of 30 V, microchannel plate detector voltage of 1600 V, desolvation temperature of 450 ^o^C, source temperature of 120 °C, cone gas flow of 50 L/h, desolvation gas flow of 550 L/h, *m/z* range of 50-1500, scan time of 0.2 s, interscan delay of 0.02 s, mode set as centroid, lockmass flow rate of 0.1 mL/min, lockmass set as leucine enkephalin (554.2615 Da) and mass accuracy window of 0.5 Da. High purity helium was used as desolvation-, cone- and collision gas. The MS analyses were set to perform unfragmented as well as four fragmenting experiments (MS^E^) simultaneously by collision energy ramping from 10 to 50 eV. Data acquisition at these various collision energies was performed to facilitate metabolite fragmentation for later assistance in downstream structure elucidation and compound annotation. Blank samples containing solvent only (50 % methanol), along with pooled biological quality controls (PBQC) were prepared. The sample run order was completely randomized with the addition of blank samples and PBQCs.

The UHPLC-ESI-MS data sets were analysed with MarkerLynx XS™ software (Waters Corporation, Manchester, UK). The MarkerLynx XS™ application uses the patented ‘ApexPeakTrack’ algorithm to perform accurate peak detection and alignment. The raw UHPLC-ESI-MS data was processed with the following parameters: 0.60–21 min retention time (Rt) range of the chromatograms and *m/z* domain of mass range 50-1500 Da. The Rts were allowed to differ by ± 0.20 min and the *m/z* values by ± 0.05 Da. The mass tolerance was 0.01 Da and the intensity threshold was 10 counts. Only the data matrices with noise level less than 50 % (MarkerLynx cut off) were retained for downstream data analyses. MarkerLynx XS performs sample normalization, based on total ion intensities of each defined peak. Prior to calculating intensities, the software performs a modified ‘Savitzky-Golay’ smoothing and integration [57–59]. Following MarkerLynx™ processing, which included noise filtering, normalization, peak detection, deisotoping and alignment [60], the generated data matrices were exported for statistical analyses.

### Multivariate data analysis and chemometric modeling

The processed UHPLC-ESI-MS data files, analysed with MarkerLynx XS™ software (Waters Corporation, Manchester, UK), were imported into SIMCA (soft independent modelling by class analogy) software, version 14.1 with activation of the ‘Omics’ skin (Sartorius Stedim Data Analytics AB, Umeå, Sweden). To put all variables on equal footing, and adjusting for measurement errors, the data was Pareto-scaled prior to chemometric modeling. A nonlinear iterative partial least squares algorithm (in-built within SIMCA software) was used to handle missing values, with a correction factor of 3.0 and a default threshold of 50 %.

Two unsupervised methods, principal component analysis (PCA) and hierarchical cluster analysis (HiCA) were applied to visualize the high-dimensional data. PCA is an unsupervised technique (similar to a clustering algorithm) that attempts to derive a set of low-dimensional features from a much larger set while still preserving as much variance as possible. Subsequently, HiCA was applied on low-dimensional data generated from the PC analyses. HiCA builds a hierarchy of clusters with an established ordering from top to bottom, with all the possible links between clusters represented by a dendrogram. Agglomerative HiCA models were computed using Ward’s linkage method (incremental sum of squares method) that considers between- and within-cluster distances when forming clusters, and the tree was sorted based on size [61]. The generated hierarchy of clusters was represented graphically as a dendrogram to evaluate whether some natural grouping emerges from the data – i.e. if the ‘metabolite space’ actually contains several distinct subspaces [13].

In addition, a supervised method, orthogonal projection to latent structures-discriminant analysis (OPLS-DA), was employed. Comparing directly multiple groups in OPLS analyses can be difficult to interpret because groups with many similar but few distinguishing characteristics may not separate. Hence, as described in the Results section, the OPLS-DA modelling was applied as a (multivariate) binary classification method to compare the control and treated samples. The OPLS-DA separates multivariate relationships into predictive (related to flagellin-treatment) and orthogonal (unrelated to the treatment) variation. This supervised method was used also for the identification of ions responsible for the discrimination between the two groups [18]. A seven-fold analysis of variance testing of cross-validated predictive residuals (CV-ANOVA) method [19] was applied as a tuning procedure in computing the supervised models [57]. The quality of the MVDA models were determined by diagnostics tools as described in the captions to the figures and in the Results section.

For variable selection, the OPLS-DA loading S-plots were evaluated. This loading plot has an S-shape provided the data are centered/Pareto-scaled, and aids in identifying variables which differ between groups (discriminating variables), i.e. variables situated at the upper right or lower left sections in the S-plot. The p1-axis describes the influence of each X-variable on the group separation (modeled covariation), and the p(corr)1-axis represents the reliability of each X-variable for accomplishing the group separation (modeled correlation). Variables that combine high model influence (high covariation/magnitude) with high reliability (i.e. smaller risk for spurious correlation) are statistically relevant as possible discriminating variables [16, 57]. However, since the S-plot is susceptible to data matrix changes due to correlation sensitivity and dependency on data structure, to avoid variable selection bias the statistical significance and discriminability of these S-plot-derived potential markers were further investigated using different tests and tools such as the variable importance in projection (VIP) plots, jackknife confidence intervals (used to estimate standard errors in a nonparametric way as an estimate of bias), variable trends and descriptive statistics. The latter were applied to selected variables, generating measures such as *p*-values, fold changes, standard deviations and CV values. VIP scoring is a metric that summarizes the importance of each variable in driving the observed group separation in a classification modelling; with the jackknife confidence intervals reflecting the variable stability. A variable with a VIP score > 1.0 means that the variable contributes more than average to the model, hence its relevance and statistically worth selecting [57].

### Metabolite annotation

The chemical/structural identities of the metabolites were elucidated using their empirical formulae obtained from the high-definition MS as well as the respective mass spectral patterns obtained during the MS^E^ analysis. MS spectral-based metabolite identification was performed based on sufficient and accurate mass fragment information, accurate calculation of the elemental composition of each discriminant feature and database searches with empirical formulae for possible metabolite annotation. MassFragment, a built in MarkerLynx XS software tool, was utilised for assigning possible structures to observed fragment ions of the precursor metabolite features using novel algorithms. The putative empirical formula of each statistically significant extracted ion peak (XIC) in the mass spectra was obtained and searched in databases for the identification of possible compound matches. These databases included ChemSpider < www.chemspider.com > , Dictionary of Natural Products < www.dnp.chemnetbase.com/ > , PubChem < http://pubchem.ncbi.nlm.nih.gov/ > , Metlin < http://metlin.scripps.edu/ > , the tomato metabolome database (MoTo) < http://www.ab.wur.nl/moto/ > , Kyoto Encyclopedia of Genes and Genomics (KEGG) Compound database < https://www.genome.jp/kegg/compound/ >  [13, 17, 23]. Metabolites were tentatively identified/annotated to level 2 of the Metabolomics Standards Initiative (MSI) [56, 62].

## Supplementary Information

**Additional file 1: Table S1.** Statistical validation of the computed OPLS-DA models corresponding to the tomato leaf elicitor treatment data matrices. **Table S2.** Annotation of discriminatory metabolites from tomato leaf tissue displaying a positive correlation towards the flagellin-derived elicitor treatments. **Figure S1.** The dynamic mechanism of M/PAMP-induced defense responses in plants. **Figure S2.** The UHPLC-MS BPI chromatograms (ESI–) of the methanolic leaf extracts from the Flg22 elicitor treated Star9001 tomato cultivar after 16 h (black), 24 h (green) and 32 h (blue). **Figure S3.** The UHPLC-MS BPI chromatograms (ESI–) of the methanolic leaf extracts from the FlgII-28 elicitor treated Star9001 tomato cultivar after 16 h (black), 24 h (green) and 32 h (blue). **Figure S4. **An orthogonal projection to latent structures discriminant analysis (OPLS-DA) model for the data processing of tomato leaf extracts of the MgSO_4_ control *vs.* Flg22 treated tissue at the 24 h incubation time

## Data Availability

The study design information, LC–MS data, data processing and analyses are reported on and incorporated into the main text.
