# Supplementary material for: Altered metabolomic states elicited by Flg22 and FlgII-28 in Solanum lycopersicum: intracellular perturbations and metabolite defenses
Source: BMC Plant Biol. 2021 Sep 21;21:429. doi: 10.1186/s12870-021-03200-5 (PMC8456652; doi:10.1186/s12870-021-03200-5)
Supplement: Supplementary file 1 — Additional file 1: Table S1. Statistical validation of the computed OPLS-DA models corresponding to the tomato leaf elicitor treatment data matrices. Table S2. Annotation of discriminatory metabolites from tomato leaf tissue displaying a positive correlation towards the flagellin-derived elicitor treatments. Figure S1. The dynamic mechanism of M/PAMP-induced defense responses in plants. Figure S2. The UHPLC-MS BPI chromatograms (ESI–) of the methanolic leaf extracts from the Flg22 elicitor treated Star9001 tomato cultivar after 16 h (black), 24 h (green) and 32 h (blue). Figure S3. The UHPLC-MS BPI chromatograms (ESI–) of the methanolic leaf extracts from the FlgII-28 elicitor treated Star9001 tomato cultivar after 16 h (black), 24 h (green) and 32 h (blue). Figure S4. An orthogonal projection to latent structures discriminant analysis (OPLS-DA) model for the data processing of tomato leaf extracts of the MgSO4 control vs. Flg22 treated tissue at the 24 h incubation time [file 12870_2021_3200_MOESM1_ESM.docx]

**Altered metabolomic states elicited by Flg22 and FlgII-28 in *Solanum lycopersicum:* intracellular perturbations and metabolite defenses**

**Dylan R. Zeiss^1^, Paul A. Steenkamp^1^, Lizelle A. Piater^1^ and Ian A. Dubery^1*^**

^1^Research Centre for Plant Metabolomics, Department of Biochemistry, University of Johannesburg, P.O. Box 524, Auckland Park 2006, South Africa.

^*^Corresponding author: idubery@uj.ac.za

**Supplementary Information**

The online version contains supplementary material available at https://doi.

**Additional file 1:**

**Table S1**. Statistical validation of the computed OPLS-DA models corresponding to the tomato leaf elicitor treatment data matrices. **Table S2**. Annotation of discriminatory metabolites from tomato leaf tissue displaying a positive correlation towards the flagellin-derived elicitor treatments.

**Figure S1**. The dynamic mechanism of M/PAMP-induced defense responses in plants. **Figure S2**. The UHPLC-MS BPI chromatograms (ESI–) of the methanolic leaf extracts from the Flg22 elicitor treated Star9001 tomato cultivar after 16 h (black), 24 h (green) and 32 h (blue). **Figure S3**. The UHPLC-MS BPI chromatograms (ESI–) of the methanolic leaf extracts from the FlgII-28 elicitor treated Star9001 tomato cultivar after 16 h (black), 24 h (green) and 32 h (blue). **Figure S4.** An orthogonal projection to latent structures discriminant analysis (OPLS-DA) model for the data processing of tomato leaf extracts of the MgSO_4_ control *vs.* Flg22 treated tissue at the 24 h incubation time.


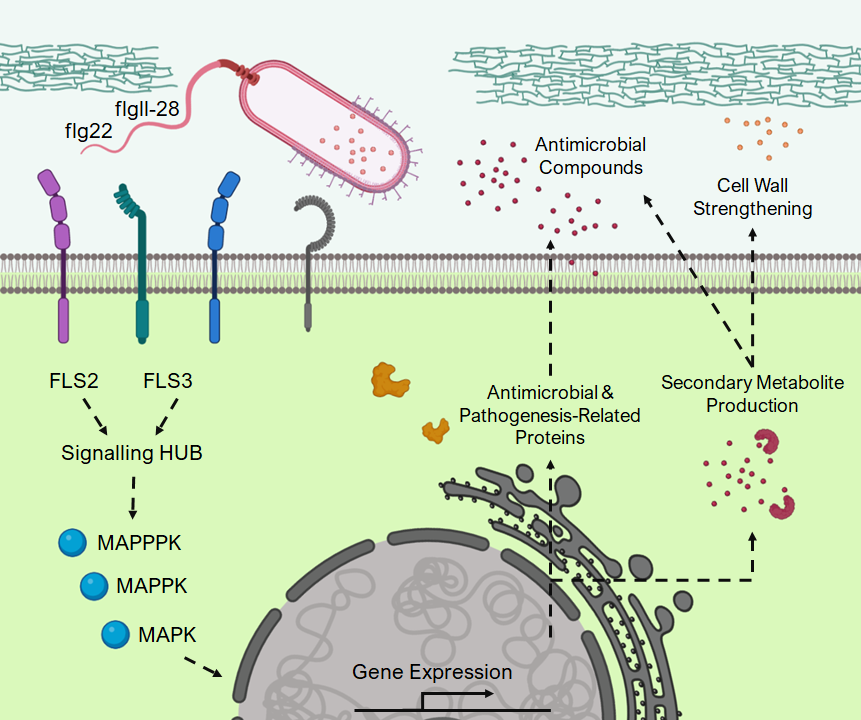


**Figure S1.** The dynamic mechanism of M/PAMP-induced defence responses in plants. The elicitor molecules, *e.g.* Flg22 and FlgII-28 are perceived by membrane bound PRRs, *e.g.* FLS2 and FLS3, leading to initial defence signalling that converges at signalling HUBS, and relays the defence signal through protein kinase cascades based on initial elicitor perception. The induced defence response then includes *R-*gene expression, the synthesis of pathogenesis-related (PR) proteins and the production of secondary metabolites that function directly (antimicrobial activity) or indirectly (radical scavenging capacity or deposition in the cell wall) in plant defence.


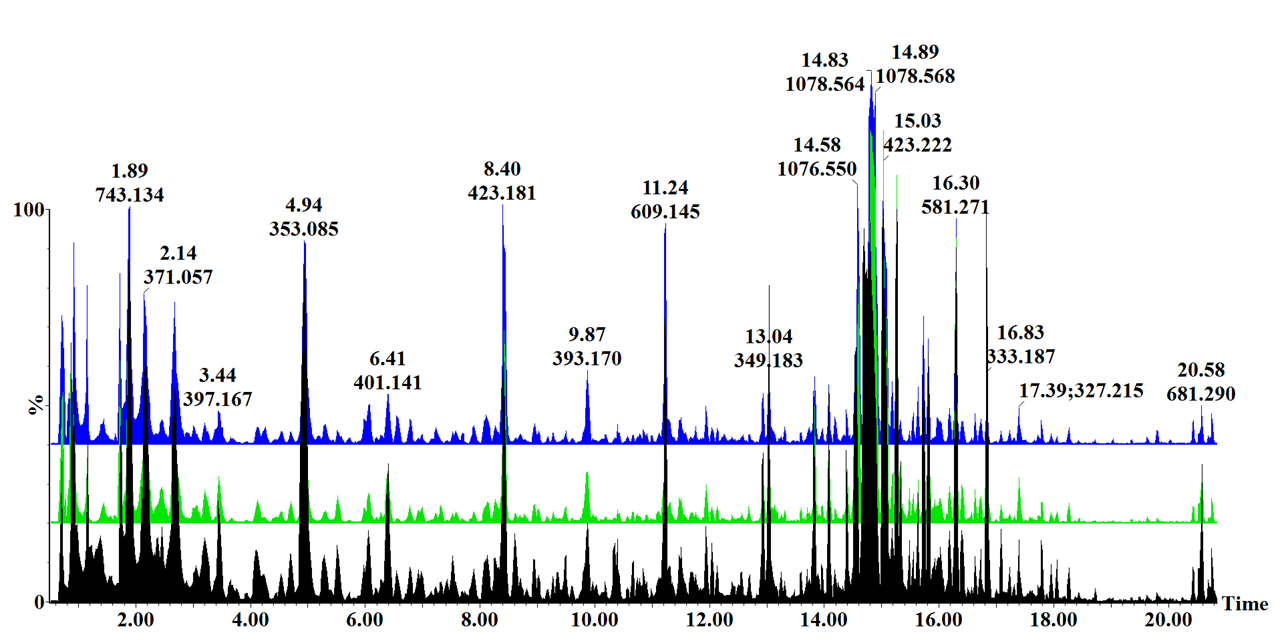


**Figure S2.** The UHPLC-MS BPI chromatograms (ESI^-^) of the methanolic leaf extracts from the Flg22 elicitor treated Star9001 tomato cultivar after 16 h (black), 24 h (green) and 32 h (blue). The chromatograms highlight metabolic variations as a result of time-dependent elicitor treatment. Qualitative differences are reflected by the peak intensities where the *y*-axis represents the relative peak intensity of the metabolites at their respective retention times (min).


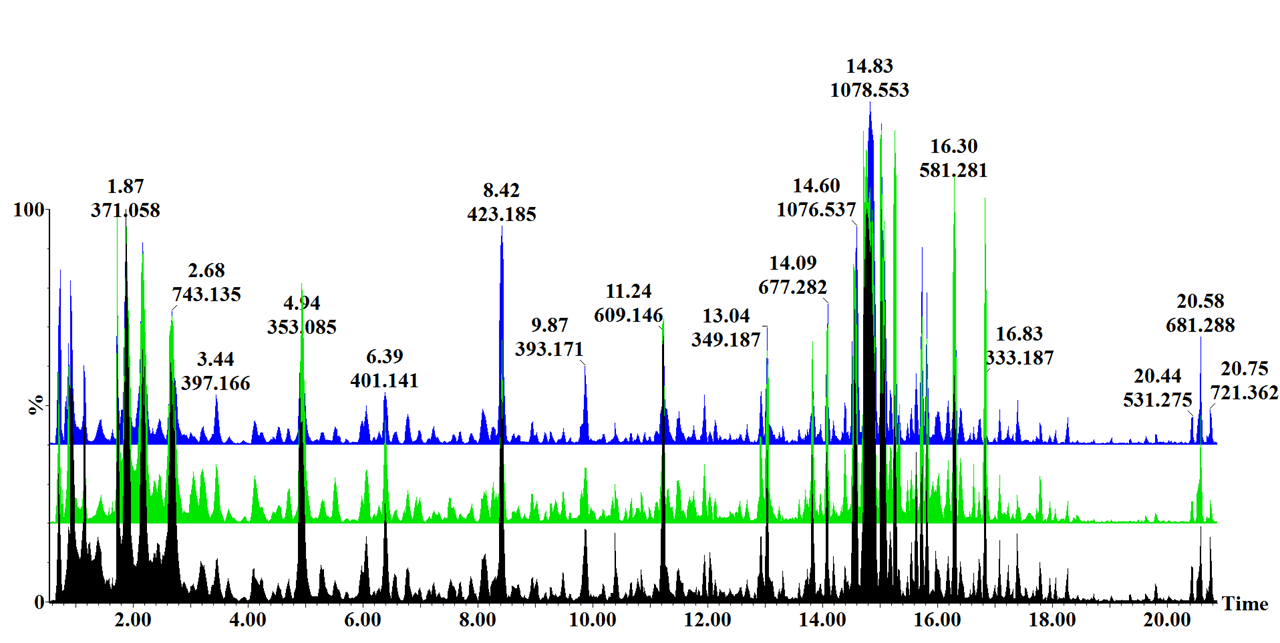


**Figure S3**. The UHPLC-MS BPI chromatograms (ESI^-^) of the methanolic leaf extracts from the FlgII-28 elicitor treated Star9001 tomato cultivar after 16 h (black), 24 h (green) and 32 h (blue). The chromatograms highlight metabolic variations as a result of time-dependent elicitor treatment. Qualitative differences are reflected by the peak intensities where the *y*-axis represents the relative peak intensity of the metabolites at their respective retention times (min).

**
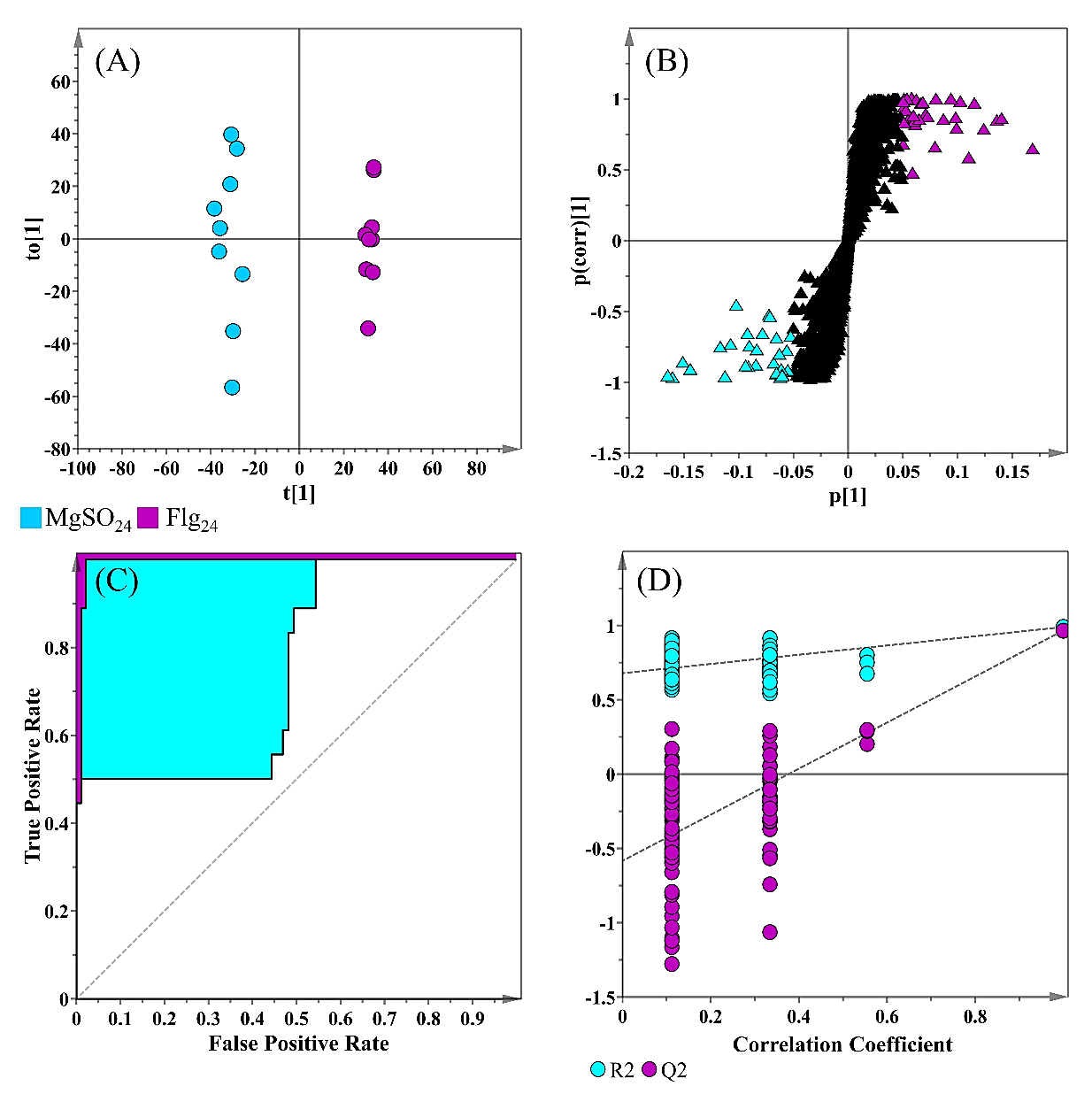
**

**Figure S4**. An orthogonal projection to latent structures discriminant analysis (OPLS-DA) model for the data processing of tomato leaf extracts of the MgSO_4_ control *vs.* Flg22 treated tissue at the 24 h incubation time. (**A**) OPLS-DA scores plot of group separation of control *vs.* treated (Flg_-24_ - purple *vs*. MgSO_4-24_ - blue) conditions. The calculated model yielded R^2^X (cum) = 53.5 %, R^2^Y (cum) = 98.9 % and Q^2^ (cum) = 96.6 %. The goodness-of-fit parameters for the OPLS model, R^2^X and R^2^Y, represent the fraction of the variance of the x and y variable explained by the model, while Q^2^Y suggests the predictive performance of the model. Model validation by 7-fold cross-validated analysis of variance (CV-ANOVA) displayed a level of statistical significance with *p*-value = 1.957 x 10^-9^. (**B**) The corresponding OPLS-DA loadings S-plot. Relevant variables at the extremes of the loadings S-plot (|p(corr)| of ≥ 0.5; |(p1)| ≥ 0.05) were selected and represent possible discriminating variables. (**C**) A receiver operating characteristic (ROC) curve summarizing the selective ability of a binary classifier (S-plot), with a classifier having perfect discrimination producing a ROC curve that passes through the top left corner to indicate 100 % sensitivity and specificity. (**D**) The response permutation test plot (*n* = 100) for the OPLS-DA model.

**Table S1.** Statistical validation of the computed OPLS-DA models corresponding to the tomato leaf elicitor treatment data matrices. The calculated R^2^X(cum), R^2^Y(cum) and Q^2^(cum) values for each of the six OPLS-DA models are presented for both ESI negative and ESI positive modes. The R^2^ and Q^2^ values of the permutation analysis (*n =* 100 random permutations) are compared and shown to be significantly lower than the original values. The *p-*value of a 7-fold CV-ANOVA was shown to indicate statistical significance of each investigated model. (MgSO = MgSO_4_ controls, Flg = Flg22, FlgII = FlgII-28; all at time intervals of 16 h, 24 h and 32 h post-elicitation).

| **Model** | **R^2^X(cum)** | **R^2^Y(cum)** | **Q^2^(cum)** | **Permutation** | | **ROC AOC** | | ***p*-value of CV-ANOVA** |
| --- | --- | --- | --- | --- | --- | --- | --- | --- |
|  |  |  |  | **R^2^** | **Q^2^** | **Control** | **Elicitor** |  |
| **ESI (Negative) Supervised Models** | | | | | | | | |
| MgSO_16_ *vs.* Flg_16_ | 0.596 | 0.997 | 0.990 | 0.685 | -0.511 | 0.993 | 0.914 | 7.065 x 10^-13^ |
| MgSO_24_ *vs*. Flg_24_ | 0.535 | 0.989 | 0.966 | 0.689 | -0.572 | 0.754 | 0.993 | 1.957 x 10^-9^ |
| MgSO_32_ *vs*. Flg_32_ | 0.432 | 0.992 | 0.967 | 0.816 | -0.386 | 0.740 | 0.582 | 1.104 x 10^-8^ |
| MgSO_16_ *vs*.  FlgII_16_ | 0.313 | 0.990 | 0.922 | 0.869 | -0.445 | 0.744 | 0.563 | 4.503 x 10^-7^ |
| MgSO_24_ *vs*.  FlgII_24_ | 0.545 | 0.992 | 0.967 | 0.719 | -0.430 | 0.643 | 0.999 | 1.636 x 10^-9^ |
| MgSO_32_ *vs*.  FlgII_32_ | 0.549 | 0.997 | 0.929 | 0.958 | -0.263 | 0.541 | 0.798 | 1.003 x 10^-5^ |
| **ESI (Positive) Supervised Models** | | | | | | | | |
| MgSO_16_ *vs.* Flg_16_ | 0.527 | 0.997 | 0.978 | 0.796 | -0.443 | 1.000 | 0.969 | 1.108 x 10^-10^ |
| MgSO_24_ *vs*. Flg_24_ | 0.663 | 0.993 | 0.984 | 0.611 | -0.494 | 0.926 | 0.875 | 1.853 x 10^-11^ |
| MgSO_32_ *vs*. Flg_32_ | 0.311 | 0.996 | 0.929 | 0.856 | -0.404 | 0.754 | 0.589 | 2.456 x 10-7 |
| MgSO_16_ *vs*.  FlgII_16_ | 0.291 | 0.994 | 0.865 | 0.894 | -0.384 | 0.841 | 0.787 | 1.482 x 10^-5^ |
| MgSO_24_ *vs*.  FlgII_24_ | 0.625 | 0.991 | 0.979 | 0.680 | -0.495 | 0.941 | 0.747 | 8.015 x 10^-11^ |
| MgSO_32_ *vs*.  FlgII_32_ | 0.470 | 0.994 | 0.901 | 0.957 | -0.335 | 0.616 | 1.000 | 6.241 x 10^-5^ |

* The goodness-of-fit parameters for the OPLS model, R^2^X, R^2^Yand Q^2^Y, were calculated which varied from 0 to 1. R^2^X and R^2^Y represent the fraction of the variance of the x and y variable explained by the model, while Q^2^Y suggests the predictive performance of the model. (Due to data size, complexity and number, other OPLS-DA models, ROC (receiver operating characteristic) curves and permutation plots are not presented, but are readily available upon request).

**Table S2**. Annotation of discriminatory metabolites from tomato leaf tissue displaying a positive correlation towards the flagellin-derived elicitor treatments

(Flg22 and FlgII-28) after 16 h (blue), 24 h (purple) and 32 h (orange) time intervals.

| **#** | **Rt**  **(min)** | ***m/z*** | **Putative identification** | **Chemical formula** | **Error (ppm)** | **Flg_16_** | **Flg_24_** | **Flg_32_** | **FlgII_16_** | **FlgII_24_** | **FlgII_32_** |
| --- | --- | --- | --- | --- | --- | --- | --- | --- | --- | --- | --- |
| 1 | 1.42 | 371.059 | Caffeoyl glucaric acid | C15H15O11 | -8.0 |  |  |  | ● |  |  |
| 2 | 3.22 | 285.058 | Genistate xylopyranoside | C12H13O8 | -12.5 | ● |  |  | ● |  |  |
| 3 | 3.45 | 397.167 | Benzoyl ornithine glycoside | C18H25N2O8 | 13.5 | ● | ● |  |  |  | ● |
| 4 | 4.13 | 658.154 | Glutathionyl-caffeoyl quinic acid | C26H32N3O15S | -1.7 |  | ● |  |  | ● |  |
| 5 | 4.70 | 431.153 | Benzyl alcohol dihexoside | C19H27O11 | -6.6 | ● |  |  |  |  |  |
| 6 | 4.95 | 353.085 | Caffeoyl quinic acid | C16H17O9 | -7.9 | ● |  | ● | ● |  |  |
| 7 | 5.30 | 353.084 | Caffeoyl quinic acid | C16H17O9 | -10.7 |  |  | ● | ● |  |  |
| 8 | 5.51 | 367.158 | Dihydroxy dimethoxy prenylchalcone | C22H24O5 | 7.9 | ● |  |  |  |  |  |
| 9 | 6.40 | 401.140 | Benzoyl alcohol pentose glc | C18H25O10 | -13.2 |  |  |  |  | ● |  |
| 10 | 6.79 | 385.110 | Sinapoyl glycoside | C17H21O10 | -10.4 |  |  |  | ● |  |  |
| 11 | 7.52 | 387.163 | Hydroxyjasmonic acid glc | C18H28O9 | -7.8 | ● | ● | ● |  |  |  |
| 12 | 8.38 | 476.155 | Unknown | C_26_H_24_N_2_O_7_ | -8.1 |  | ● | ● |  | ● |  |
| 13 | 8.63 | 367.100 | Feruloyl quinic acid | C17H20O9 | -9.3 | ● |  |  | ● |  | ● |
| 14 | 9.37 | 296.061 | Benzoyl oxindole acetic acid | C17H14NO4 | -10.9 | ● |  |  | ● | ● |  |
| 15 | 9.86 | 245.090 | Acetyl tryptophan | C13H13N2O3 | -12.9 |  | ● |  |  | ● | ● |
| 16 | 10.35 | 344.112 | Feruloyl noradrenaline | C18H18NO6 | -5.6 | ● |  |  | ● | ● |  |
| 17 | 10.74 | 298.107 | Coumaroyl dopamine | C17H17NO4 | -4.9 |  |  | ● | ● |  |  |
| 18 | 10.99 | 444.165 | Coumaroyl tyramine glc | C17H17NO3 | 20.6 |  |  | ● |  |  | ● |
| 19 | 11.20 | 460.160 | Unknown | C_23_H_26_NO_9_ | -2.8 |  |  | ● |  | ● | ● |
| 20 | 11.21 | 609.145 | Rutin | C27H30O16 | -1.8 | ● |  | ● | ● |  |  |
| 21 | 11.75 | 490.170 | Feruloyl dopamine glc | C20H29NO13 | 27.3 |  |  | ● |  | ● | ● |
| 22 | 12.97 | 328.117 | Feruloyl dopamine | C15H20NO8 | 1.9 |  |  |  | ● | ● | ● |
| 23 | 13.00 | 349.183 | Acetyl feruloyl agmatine | C17H25N4O4 | -14.6 | ● |  |  |  | ● |  |
| 24 | 13.73 | 282.112 | Coumaroyl tyramine | C17H16NO3 | -5.5 |  |  | ● | ● |  | ● |
| 25/26 | 13.81 | 677.282 | Unknown | C_27_H_49_O_19_ | -7.8 |  | ● |  |  | ● | ● |
| 27 | 14.20 | 312.121 | Feruloyl tyramine | C18H18NO4 | -10.0 |  |  |  | ● |  |  |
| 28 | 14.40 | 453.231 | PG (14:1(9Z)/0:0) | C20H38O9P | 11.2 | ● |  |  |  |  |  |
| 29 | 14.59 | 1076.520 | Dehydrotomatine + FA | C51H82NO23 | -7.7 |  |  |  |  | ● |  |
| 30 | 14.83 | 1078.560 | α-Tomatine + FA | C51H83NO23 | 14.8 |  | ● | ● |  | ● |  |
| 31 | 15.02 | 423.184 | PA (8:0/8:0) | C19H37O8P | 15.7 |  | ● |  |  | ● |  |
| 32 | 15.20 | 447.220 | Unknown | C_21_H_35_O_10_ | -7.9 |  | ● |  |  | ● |  |
| 33 | 15.25 | 423.221 | PA (8:0/8:0) | C19H37O8P | 13.4 | ● |  |  |  | ● |  |
| 34 | 15.32 | 495.255 | Palmitoyl-glycero-PS | C22H43NO9P | -14.3 | ● |  |  |  |  |  |
| 35 | 16.25 | 495.257 | Palmitoyl-glycero-PS | C22H43NO9P | -16.6 |  |  | ● |  |  | ● |
| 36 | 16.30 | 581.281 | PI (17:2(9Z.12Z)/0:0) | C26H46O12P | 13.3 | ● |  |  |  | ● |  |
| 37 | 16.72 | 327.214 | Trihydroxyoctadecadienoic acid | C18H31O5 | -11.2 |  | ● |  |  |  |  |
| 38 | 16.82 | 333.188 | Hydroxydecanoic acid rhamnoside | C16H29O7 | -11.6 | ● |  |  |  |  |  |
| 39 | 17.41 | 329.231 | Trihydroxyoctadecenoic acid | C18H33O5 | -7.1 |  | ● |  | ● |  |  |

Metabolites were annotated in ESI(–) mode using ultra-high performance liquid chromatography coupled to high definition mass spectrometry (UHPLC–MS). The metabolite features were annotated according to level 2 of the Metabolomics Standards Initiative (MSI, [56]). * Abbreviations: glc = glycoside, PS = phosphoserine, PI = phosphatidyl inositol, PA = phosphatidic acid, PG = phosphatidyl glycerol, FA = formic acid adduct.
